# Supplementary material for: Saturated Iso-Type Fatty Acids from the Marine Bacterium Mesoflavibacter zeaxanthinifaciens with Anti-Trypanosomal Potential
Source: Pharmaceuticals (Basel). 2024 Apr 13;17(4):499. doi: 10.3390/ph17040499 (PMC11053438; doi:10.3390/ph17040499)
Supplement: Supplementary file 1 [file pharmaceuticals-17-00499-s001.zip › pharmaceuticals-2901792-supplementary.pdf]

SUPLEMENTARY MATERIAL

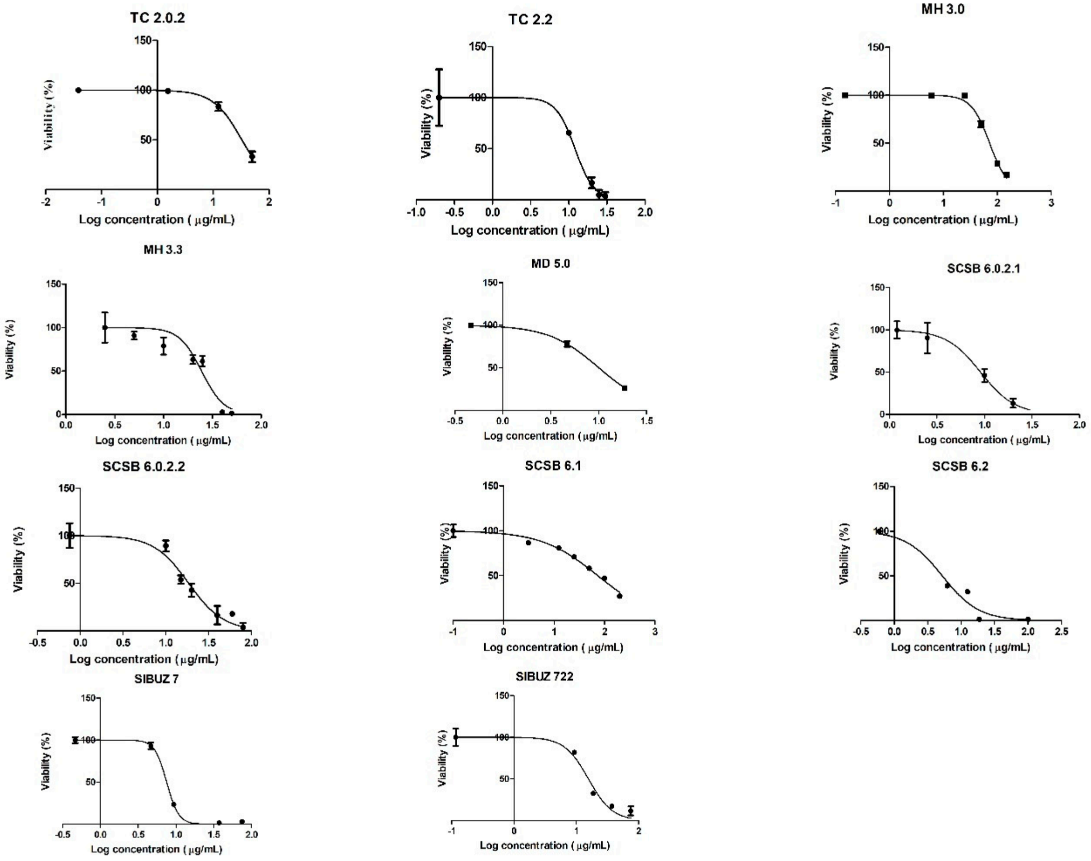

Figure S1-Dose-response curves of microbial metabolites against *T. cruzi* trypomastigotes. TC (*Tubastraea coccinea*), MH (*Mussismilia hispida*), MD (*Madracis decactis*), SCSB (Sediments of the São Sebastião Channel), SIBUZ (Sediments of Buzios Island).

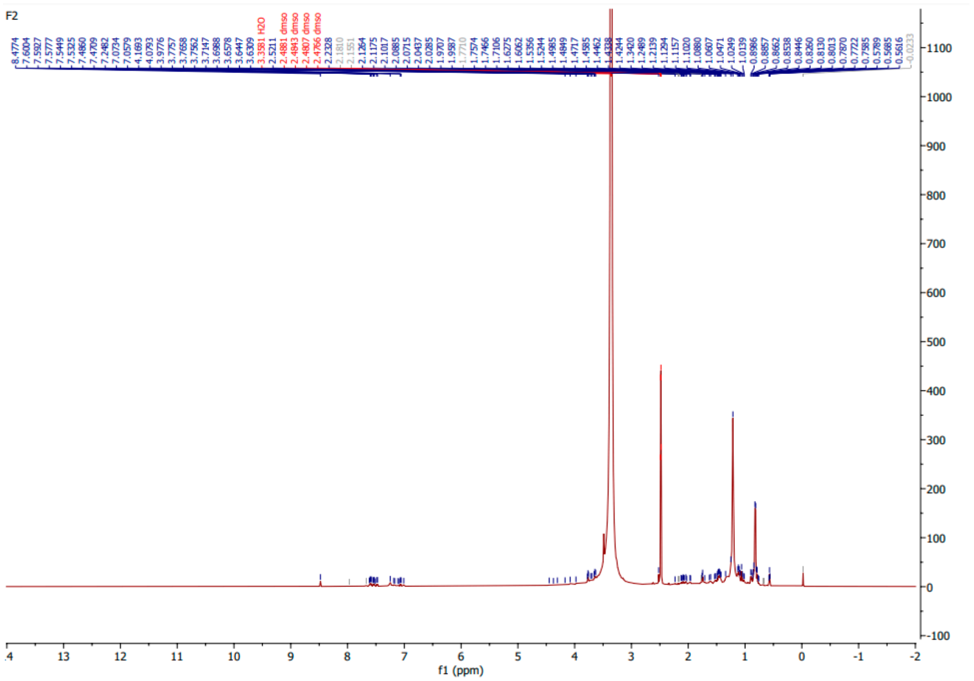

Figure S2 A– NMR ^{1}H espectrum of fraction II from *M. zeaxanthinifaciens* EtOAc extract (FII).

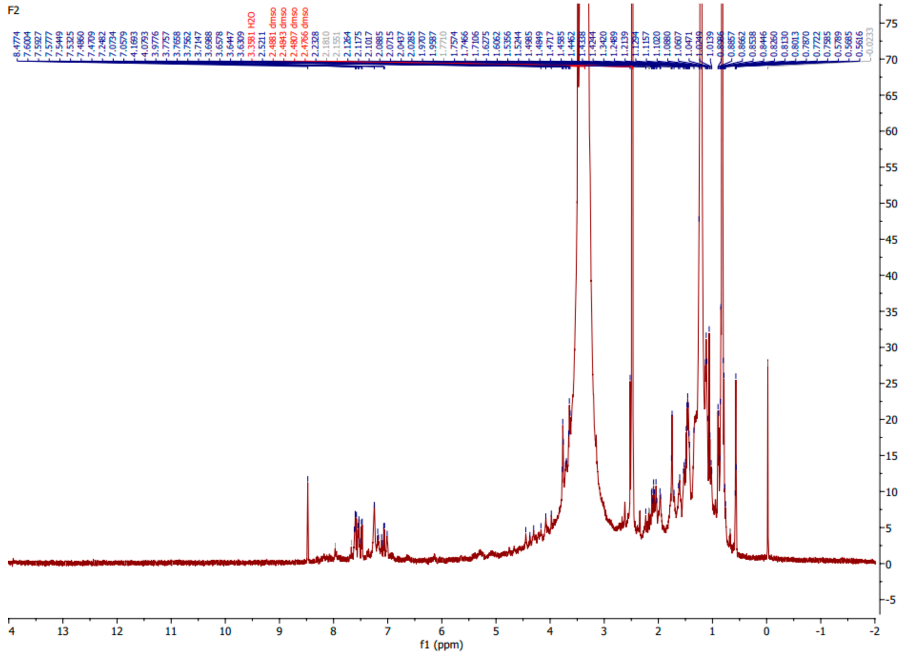

Figure S2 B – NMR ¹H espectrum of fraction II from *M. zeaxanthinifaciens* EtOAc extract (FII).

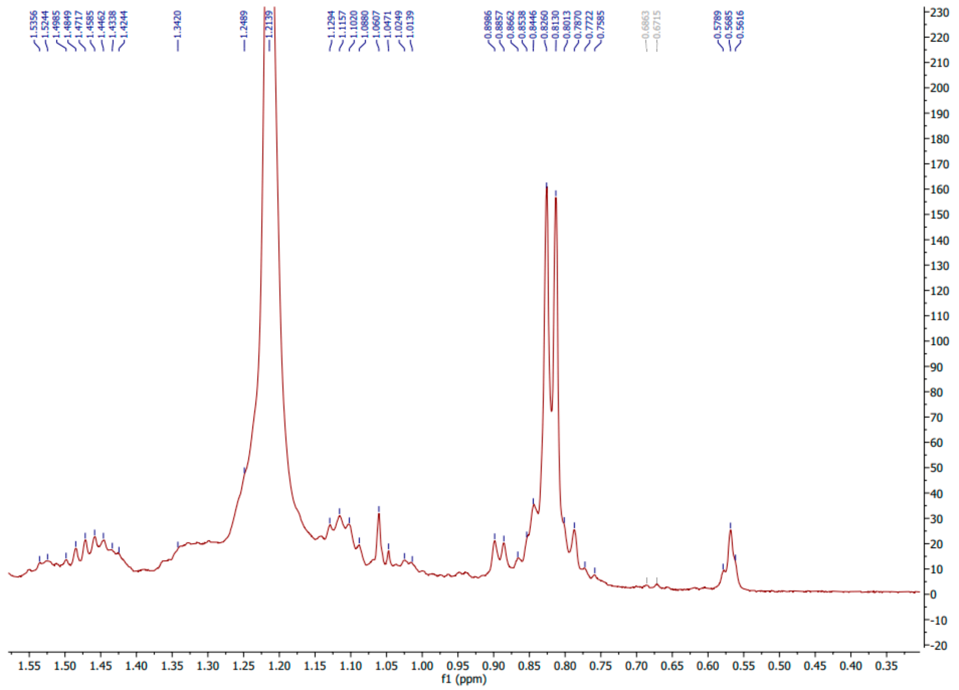

Figure S2 C– NMR ¹H espectrum of fraction II from *M. zeaxanthinifaciens* EtOAc extract (FII).

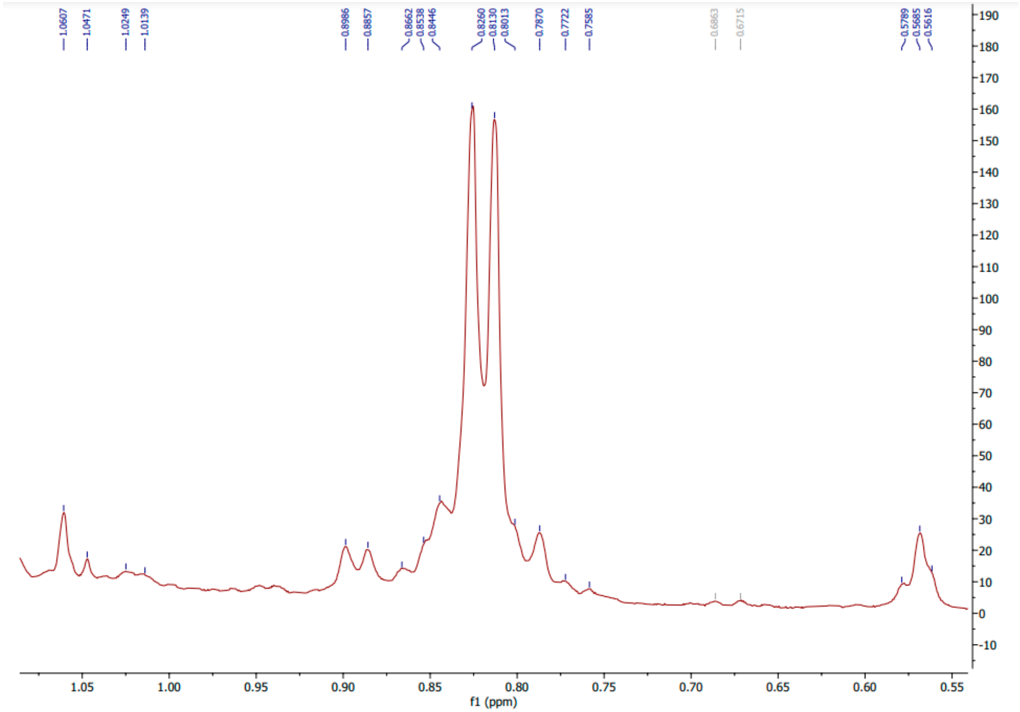

Figure S2 D– NMR ¹H espectrum of fraction II from *M. zeaxanthinifaciens* EtOAc extract (FII).

Table S1- Protein signals (m/z) in MALDI-TOF/MS of trypomastigotes of *T. cruzi* in the presence of fraction II from *M. zeaxanthinifaciens* EtOAc extract (FII). Trypomastigotes were treated with FII at 25 and 50 µg/mL, and benznidazole (BZN-40 µM) was used as standard drug. Parasites without treatment were used as control (untreated). The parasites were treated for 24 h and the protein profile was compared to untreated parasites.

BZN 40 μM				FII 25 μg/mL				FII 50 μg/mL				Control			
m/z	Res.	Intens.	Area	m/z	Res.	Intens.	Area	m/z	Res.	Intens.	Area	m/z	Res.	Intens.	Area
2664,075	1066	2519	5613	2015,834	743	2075	4187	2691,17	904	4531	10087	2015,135	642	2350	4703
2682,109	1386	2118	3449	2056,46	845	4410	11809	2781,908	694	4287	11698	2055,506	673	4897	15525
2690,333	672	3805	13954	2063,342	690	1764	3954	3483,932	1095	4161	7632	2068,946	581	2273	6339
2699,407	1014	2970	7126	2070,153	913	2027	4642	3577,487	497	4478	10515	2335,717	594	3180	10503
2781,947	729	2214	4701	2327,608	540	1871	4262	3613,278	583	4274	11884	2689,354	1349	2573	4436
3578,838	819	3309	11560	2336,717	567	2824	9740	3627,547	686	4440	11838	2696,163	525	2381	7139
3979,141	654	2850	14765	2690,359	1170	2586	5468	3977,528	852	8587	41946	2780,356	580	6699	32802
3984,344	1677	2485	6411	2698,036	599	2530	9427	3998,382	1121	6298	25167	2896,711	577	3940	17374
3997,886	838	7370	53474	2781,729	588	6872	32379	4006,558	2883	4686	5541	2966,007	748	2967	8942
4011,094	1096	4074	24163	2898,119	564	3458	16230	4009,553	2241	4814	6482	3173,896	745	2659	6899
4024,91	895	4605	29836	2966,813	542	2100	7688	4013,901	2809	4880	8414	3577,343	630	5032	23540
4038,159	808	13807	86745	3175,138	904	1891	3974	4023,769	772	5766	21999	3771,764	597	4507	20408
4052,022	699	6394	50155	3578,712	762	3907	14653	4038,211	904	14855	76509	3947,291	660	4561	23072
4063,586	3257	3165	7429	3599,472	775	2401	6816	4052,234	1359	7104	33371	3956,013	1666	2427	3751
4070,523	1075	2802	9824	3773,812	761	5251	24416	4068,325	999	5311	18122	3961,779	1572	2555	5176
4078,051	755	3374	26298	3948,816	682	3895	20333	4077,888	3275	4525	7379	3975,89	750	15257	92591
4090,429	2793	2669	5838	3960,786	1146	2151	5822	4087,562	1229	4398	9575	3987,679	1267	2835	4551
4101,786	2362	2408	6320	3977,658	737	15163	95745	4117,196	793	6537	31489	3997,246	901	4335	27493
4112,01	862	2197	6445	3999,67	856	4262	20001	5656,149	792	2999	7757	4004,478	1633	3411	8242
4116,162	1118	2699	9873	4006,182	922	3398	17659	6143,043	865	2837	16054	4010,492	1162	3280	9356

4130,501	1105	2070	5795	4017,218	1339	3262	9436	6161,906	875	2670	9033	4021,161	491	3291	19935
4401,734	723	1835	4872	4023,219	1015	3803	23828	6602,605	1564	2531	7102	4021,161	506	3291	19935
4407,563	1708	1968	3065	4038,038	817	12814	74765	7966,312	1102	1621	7122	4036,34	733	10951	74389
5035,447	629	1779	7855	4050,989	742	5061	36149	7980,687	1165	1582	6373	4050,957	792	4319	27099
6603,142	786	2376	25165	4065,327	800	3362	18127	7995,453	1025	3665	31565	4063,556	1161	3597	12286
6622,662	1264	1546	5755	4074,106	564	3244	20770	8008,324	3788	1859	7294	4067,144	2727	3542	6884
6668,455	690	1440	6961	4091,268	722	2469	10014	8022,537	842	2152	13304	4072,719	609	4142	22853
6674,031	2359	1277	2303	4100,965	700	2568	10380	8025,856	843	2106	13304	4085,751	1461	3376	9325
6698,247	862	1290	5423	4117,199	669	15808	109194	8046,852	960	2935	23453	4089,843	556	3361	13147
6709,741	589	2052	20807	4131,566	751	4679	30309	8060,908	1401	2482	12419	4100,177	968	3356	12341
6727,793	1865	1328	4336	4145,062	771	6235	43857	8074,721	1099	9983	93513	4115,588	704	18291	132043
6735,048	780	1279	4454	4156,108	1600	2475	4461	8088,456	1557	3400	19755	4129,801	798	5572	33200
7954,533	1085	1086	5248	4161,836	522	2337	9511	8101,083	721	3701	37895	4143,516	796	6462	45114
7968,452	1315	1753	14646	4185,037	551	2070	6884	8116,38	1435	2226	11907	4156,627	2711	2738	5112
7981,873	2625	1552	6528	4399,737	606	2001	6564	8125,179	3113	2016	9935	4399,958	937	2366	6558
7995,695	1151	6660	61530	5307,851	842	1601	7211	8137,872	1881	1767	5941	4597,734	2856	2194	4904
8009,644	1568	2156	13194	5435,754	571	1571	8767	8146,327	4957	1689	3797	5434,395	719	2441	14482
8024,751	1245	2869	30468	5436,844	583	1638	8767	8156,47	1407	1620	5447	5526,855	1088	2266	12774
8033,958	2369	2207	9796	6505,132	1089	1245	5287	8171,721	876	2199	20891	5547,229	867	3100	22565
8046,276	1256	3499	38357	6602,089	1171	2391	24599	10065,73	3366	749	3033	5561,44	3342	2024	4402
8062,036	1616	2793	14850	6709,019	1840	1265	3488					6130,69	1262	3077	18547
8075,276	1069	11741	124173	7980,3	1512	868	3384					6137,735	1865	2601	8617
8088,679	1706	3856	26886	7995,48	1084	2298	20130					6142,914	1659	2344	7341
8102,604	1132	4771	70224	8011,836	864	1030	7340					6148,784	1599	2172	5678
8125,648	1129	2015	11388	8015,26	860	1042	7340					6174,786	2025	1749	4143
8136,189	1072	1953	12847	8024,211	1275	1232	7064					6197,242	1354	1751	5097
8142,095	1703	1940	9101	8036,255	1381	1130	5271					6599,239	883	2510	20758
8152,799	839	1986	20633	8047,961	1097	1854	16816					6703,924	770	2049	11681

8157,555	809	1932	20633
8165,219	3272	1692	6707
8181,083	2050	1565	9282
8192,744	1351	1123	6818
8215,414	1094	1070	5516
8225,605	2028	946	3339
8241,23	805	1042	8116
8246,196	758	988	8116
8261,63	965	949	5386
10045,72	1675	561	2760
10056,49	3465	853	4149
10064,82	3387	957	6353
10074,89	2555	900	5667
10085,55	4243	624	2340
10095,25	2230	606	3042
13199,85	1554	370	4883
13221,16	3172	315	2023

8060,33	2059	1453	6464
8075,139	965	5797	59593
8089,911	963	1984	15474
8101,691	959	2397	23102
8113,748	1260	1253	11769
8131,963	4342	1006	2655
8148,016	5531	970	3572
8153,875	4106	894	2747
10063,57	1037	537	3623
13202,43	1738	512	4691
13220,56	4840	287	1512

6712,014	772	1702	7084
6894,768	1364	2213	13286
6909,344	532	2058	16285
7992,107	1129	1780	19647
8020,221	935	1242	8958
8045,452	874	1534	13423
8071,967	949	4623	52911
8087,243	1039	1686	10977
8099,025	1082	1896	17291
10061,43	1690	712	2575
11051,53	1239	669	6785
11092,3	1197	841	11463
12257,4	1169	658	8532
12270,45	5005	600	2565
12275,62	1931	491	2791
12285,04	2430	500	2828
12299,2	3741	431	1765
12312,08	5862	407	1942
13199,45	1394	510	4705
13789,34	2094	394	3385
13807,86	3978	327	1678
13829,91	4910	314	1084
13839,86	1816	314	1831
